# Interpreting protein abundance in *Saccharomyces cerevisiae* through relational learning

**DOI:** 10.1093/bioinformatics/btae050

**Published:** 2024-01-25

**Authors:** Daniel Brunnsåker, Filip Kronström, Ievgeniia A Tiukova, Ross D King

**Affiliations:** Department of Computer Science and Engineering, Chalmers University of Technology, Gothenburg 412 96, Sweden; Department of Computer Science and Engineering, Chalmers University of Technology, Gothenburg 412 96, Sweden; Department of Life Sciences, Chalmers University of Technology, Gothenburg 412 96, Sweden; Department of Industrial Biotechnology, KTH Royal Institute of Technology, Stockholm 106 91, Sweden; Department of Computer Science and Engineering, Chalmers University of Technology, Gothenburg 412 96, Sweden; Department of Chemical Engineering and Biotechnology, University of Cambridge, Cambridge CB3 0AS, United Kingdom; The Alan Turing Institute, London NW1 2DB, United Kingdom

## Abstract

**Motivation:**

Proteomic profiles reflect the functional readout of the physiological state of an organism. An increased understanding of what controls and defines protein abundances is of high scientific interest. *Saccharomyces cerevisiae* is a well-studied model organism, and there is a large amount of structured knowledge on yeast systems biology in databases such as the Saccharomyces Genome Database, and highly curated genome-scale metabolic models like Yeast8. These datasets, the result of decades of experiments, are abundant in information, and adhere to semantically meaningful ontologies.

**Results:**

By representing this knowledge in an expressive Datalog database we generated data descriptors using relational learning that, when combined with supervised machine learning, enables us to predict protein abundances in an explainable manner. We learnt predictive relationships between protein abundances, function and phenotype; such as α-amino acid accumulations and deviations in chronological lifespan. We further demonstrate the power of this methodology on the proteins His4 and Ilv2, connecting qualitative biological concepts to quantified abundances.

**Availability and implementation:**

All data and processing scripts are available at the following Github repository: https://github.com/DanielBrunnsaker/ProtPredict.

## Graphical abstract

**Graphical abstract:**
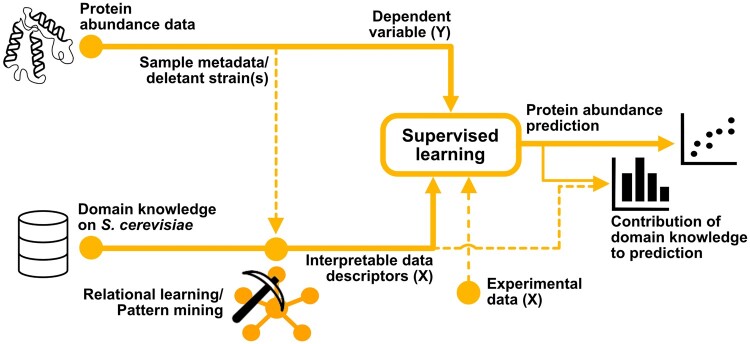

## 1 Introduction

Understanding how gene deletions or changes in gene content affect biological readouts is a key question in systems biology, synthetic biology, and biotechnology (Benfey and Mitchell-Olds 2008). In the most well-studied of organisms, such as the yeast *Saccharomyces cerevisiae*, we have functional annotations for a majority of genes but exhaustive understanding of regulatory rules at a systems level remains a challenge (Wood *et al.* 2019).

As a model organism, the scientific community has systematized a substantial amount of information on *S.cerevisiae* systems biology, often in the forms of vast online databases. This includes databases such as the Saccharomyces Genome Database (SGD), BioGRID, and metabolic models such as Yeast8 (Cherry *et al.* 2012, Lu *et al.* 2019, Oughtred *et al.* 2021). In these databases, there exists information in a multitude of different data modalities, containing findings and metadata on thousands of experiments represented in a semantically rich form. This makes it suitable for relational machine learning, in which one can learn complex patterns across the content of the database using pattern mining, with the intention of uncovering biologically relevant regulatory rules (King *et al.* 2001, Raedt 2008, Muggleton *et al.* 2012, Orhobor *et al.* 2020). Additionally, recent advances in proteomics have led to the generation of protein profiles which has been used to further increase our understanding of yeast systems biology and what drives protein abundances (Messner *et al.* 2022; Messner  *et al.* 2023).

A crucial aspect in applying machine learning techniques is how to properly represent data in a meaningful way. While popular methods such as deep neural networks are very successful in extracting rich internal representations from data, they tend to have poor interpretability. They also tend to make poor use of existing domain knowledge without specifically encoding it in the structure (Zhou *et al.* 2020, Zhang *et al.* 2021). In contrast, there are other machine learning ways to generally represent data in a structured manner that enables one to account for background domain knowledge. The richest way to represent background knowledge are logic programs. These programs, using a subset of first-order predicate logic, are very flexible and can represent different types of data in one formalism, and allows for an explicit way of representing relations between entities (Lloyd 2012). The use of logic programs as a target for machine learning is known as “relational learning” or “inductive logic programmming” (Raedt 2008).

Propositionalization is a machine learning methodology in which one extracts tabular/propositional data from relational databases or logic programs (Kramer *et al.* 2001, Lavrač *et al.* 2020). By transforming a relational into a propositional representation, we can make use of learning methods in a relational setting, while also partially negating one of its main disadvantages; computational inefficiency. This is achieved by using the much more efficient attribute-value based learners we use today, such as support vector machines and gradient boosting to learn the predictive model itself (Orhobor *et al.* 2020).

Here, we combine several of these complex relational databases to construct a highly expressive Datalog database. We performed relational learning over the content of this database and generated binary descriptors through propositionalization, which were then directly processed by standard machine learning algorithms. These descriptors are represented in the form of logic programs, which can be translated into statements interpretable by domain experts. By combining these descriptors with propositional data, such as the protein abundances generated by Messner *et al.* (2023) we can use techniques in model explainability to evaluate and (to some extent) quantify the contribution of regulatory interactions, phenotypical markers and current functional annotations of the genotype. We found that this methodology provides informative and interpretable features for domain experts. The resulting models could also be used to guide metabolic engineering strategies and infer protein function.

## 2 Materials and methods

### 2.1 Relational database of yeast systems biology

The database makes use of the Saccharomyces Genome Database (SGD), BioGRID, and Yeast8 as sources of relational data (Cherry *et al.* 2012, Lu *et al.* 2019). All of the data used to create the database were downloaded using either YeastMine (retrieved 16 December 2022) or the Metabolic Atlas (retrieved 17 December 2022) (Balakrishnan *et al.* 2012, Wang *et al.* 2021, Li *et al.* 2023). The database consists of relations covering metabolism, function, structure, regulation, and phenotype. Additional details regarding the relations can be found in Supplementary Notes SI.

For the gene-ontology terms, “manually curated,” “high-throughput,” and “computational” GO annotations were included. The dataset was curated, as to remove observations with a different strain-background than the one used in the dataset by Messner *et al.* (2023) (S288c). Invalid GO-terms and dose-dependent phenotypes, such as toxin resistance and resistance to chemicals were also removed. Gene-to-metabolite relations from Yeast8 that involved very common reactants and products unlikely to contribute to metabolic regulation (such as $H^{+}$ and $H_{2}O$) were removed from the database, as defined in Waller *et al.* (2020).

The data were then represented in Datalog, a declarative programming language which allows for richer representations of relational knowledge than what is possible in standard relational database query languages such as SQL (Dehaspe and Toivonen 1999).

### 2.2 Frequent pattern mining and dataset construction

In order to extract biologically relevant patterns from the constructed database, relational learning was applied in the form of frequent pattern mining. These patterns were learned using the *induce features* mode in the ILP-engine aleph (version 5, https://www.cs.ox.ac.uk/activities/programinduction/Aleph/aleph.html), utilizing a simplified version of the data-mining algorithm WARMR to find frequent relational patterns using sample meta-data (deletant strain) as positive examples (Dehaspe and Toivonen 1999, Srinivasan 2001). WARMR is an ILP data mining algorithm that makes use of a level wise search algorithm that searches in order of pattern-generality, starting from the most general patterns (or simple instantiations of facts) and iteratively adds logical conditions until a predefined level of specificity has been reached (King *et al.* 2001). The goal of the algorithm in this case is to define valid patterns with as high coverage of positive examples as possible, given the following constraints: minimum allowed fraction of positive examples in a single clause at 0.025%, maximum amount of features 2048, the upper bound of new variable layers to 10, and with an allowed clause-length of 10. All of the aforementioned steps were performed in Prolog (SWI-Prolog, v7.6.3) (Wielemaker *et al.* 2012). All relations directly derived from the datasets used as predictive targets were removed prior to pattern mining. The type of data, allowed relations, along with a summary of their biological relevance can be seen in Supplementary Notes I. Note that the search was restricted to a subset of first-order logic, due to the inherent difficulties of propositionalizing higher-order logic statements.

**Figure 1.**
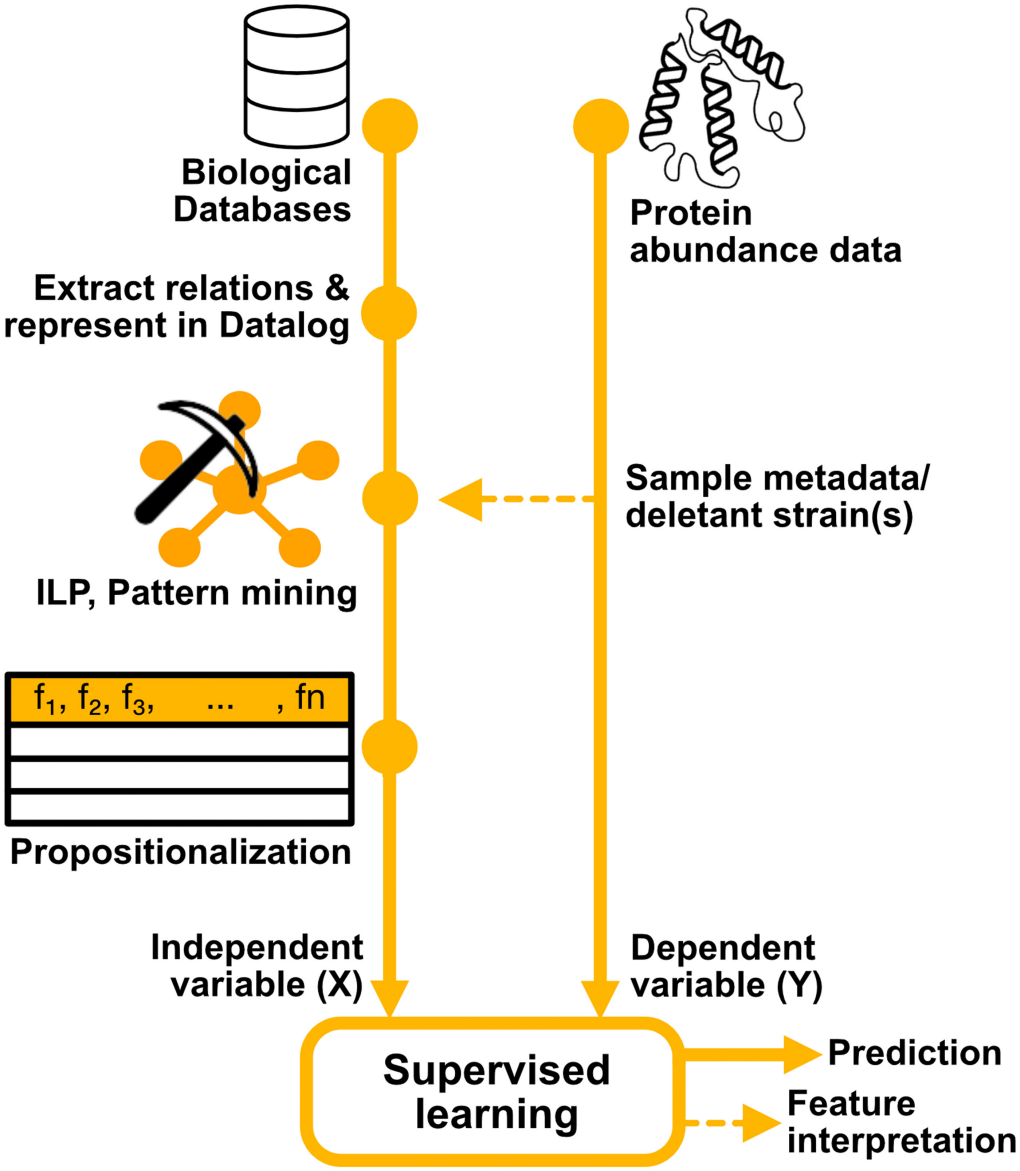
Dataset construction using frequent pattern mining on databases on yeast systems biology, utilizing sample meta-data from a proteomics dataset by Messner *et al.* (2022). Biological databases are represented in Datalog. WARMR is utilized (using sample meta-data from the selected dataset) to extract frequent patterns from the database. These patterns are propositionalized and used as independent variables (predictive variables) in the prediction of protein abundances.

A proteomic dataset was previously generated by Messner *et al.* (2023), in which profiles consisting of thousands of proteins were measured for ∼4600 nonessential gene deletions. The mined, propositionalized, patterns were then combined with this protein abundance data [or amino acid concentration data from Mülleder *et al.* (2016)] by merging on the ORF (open reading frame), describing the positive example in the case of the relational dataset, and the deletant strain in the case of protein abundance data. The patterns were used as predictive features (independent variables) and all of the different abundance values (per protein) were then used as dependent variables in regression. A visual summary of this can be seen in Fig. 1.

The propositional descriptors for each the available deletant strains were then saved as tabular datasets (with accompanying explanatory logic programs). More detailed instructions, along with the generated descriptors, can be found at https://github.com/DanielBrunnsaker/ProtPredict.

### 2.3 Training and evaluation

The scikit-learn implementation of XGBoost (v1.6.1) was used as the model of choice for all regression tasks in this study, as it is a very efficient and highly performant machine learning algorithm (Chen and Guestrin 2016). It also performed favourably when compared to other supervised learning algorithms (see Supplementary Notes SVI). The hyper-parameters were tuned on the first example protein in the dataset (A5Z2X5/Min8/YPR010C-A) with cross validated Bayesian optimization, using sci-kit optimize (v0.9.0). All of the models (predicting abundances for separate proteins) were evaluated using 5-fold cross validation (CV). This type of evaluation procedure was chosen as interpretability across the whole dataset was of main concern, and making sure that informative features were added to the prediction task was of higher importance. As such, an independent test-set was deemed unnecessary for this specific purpose (King *et al.* 2021). The metrics used for training and evaluation were *MSE* (mean squared error) and $R^{2}$ (coefficient of determination) respectively.

### 2.4 Feature importance

Features (α-amino acids and binary data descriptors) were analyzed using a model agnostic feature importance tool, SHAP (SHapley Additive exPlanations) and an XGBoost-specific one, gain. Gain describes the relative contribution of a feature to the model by calculating the improvement in performance the feature brings (Chen and Guestrin 2016). SHAP is based on a game-theoretic approach and is used to evaluate the outputs of any machine learning model (Lundberg and Lee 2017). It connects optimal credit allocation with local explanations by estimating instance-wise Shapley values. More specifically, TreeExplainer, a SHAP-based methodology optimized for tree-based machine learning models, was used (Lundberg *et al.* 2020).

## 3 Results

### 3.1 Frequent pattern mining

As seen in Fig. 1, a database was constructed by establishing gene-to-entity relations from SGD, BioGRID, and Yeast8 (Cherry *et al.* 2012, Lu *et al.* 2019, Oughtred *et al.* 2021). These relations were then described decalaratively in Datalog (Dehaspe and Toivonen 1999). This relational database was then used as a basis for frequent pattern searches using a simplified version of the WARMR-algorithm implemented in Aleph, utilizing sample meta-data (deletetant strains) as positive examples (Srinivasan 2001). The patterns were then represented as logic programs (Dehaspe and Toivonen 1999, King *et al.* 2001, Srinivasan 2001). An example of a typical pattern (biological concept), represented as a Prolog program is:


$$\begin{array}{c} \mathrm{Gene(A):}=\\ \;\;\mathrm{RegulatedBy(A,}\;\mathrm{B,}\;\mathrm{Transcription}\;\mathrm{factor),}\\ \;\;\mathrm{nullPhenotype(B,}\;\mathrm{Abnormal}\;\mathrm{chronological}\;\mathrm{lifespan),}\\ \;\;\mathrm{InvolvedIn(A,}\;\mathrm{One—carbon}\;\mathrm{metabolic}\;\mathrm{process)} \end{array}$$

This program can be interpreted as: genes (A) which are involved in the one-carbon metabolic process, and which are regulated by a transcription factor (B) whose deletion causes the cell to have an abnormal chronological lifespan.

### 3.2 Protein abundance can be predicted directly from relational data descriptors

The generated programs (descriptors) were used as predictive features (independent variables) for the prediction of abundances for all of the 2292 proteins present in the dataset generated by Messner *et al.* (2023). Feature importances were calculated using both a model agnostic method, SHAP, and gain, a model specific importance metric (Chen and Guestrin 2016, Lundberg and Lee 2017, Lundberg *et al.* 2020). To assess the predictive capability of the features across the whole space of predictable proteins, values were normalized and averaged across the span of all the trained models. All of the presented patterns along with translations into English are given in the Supplementary Notes (SII–SV).

**Figure 2.**
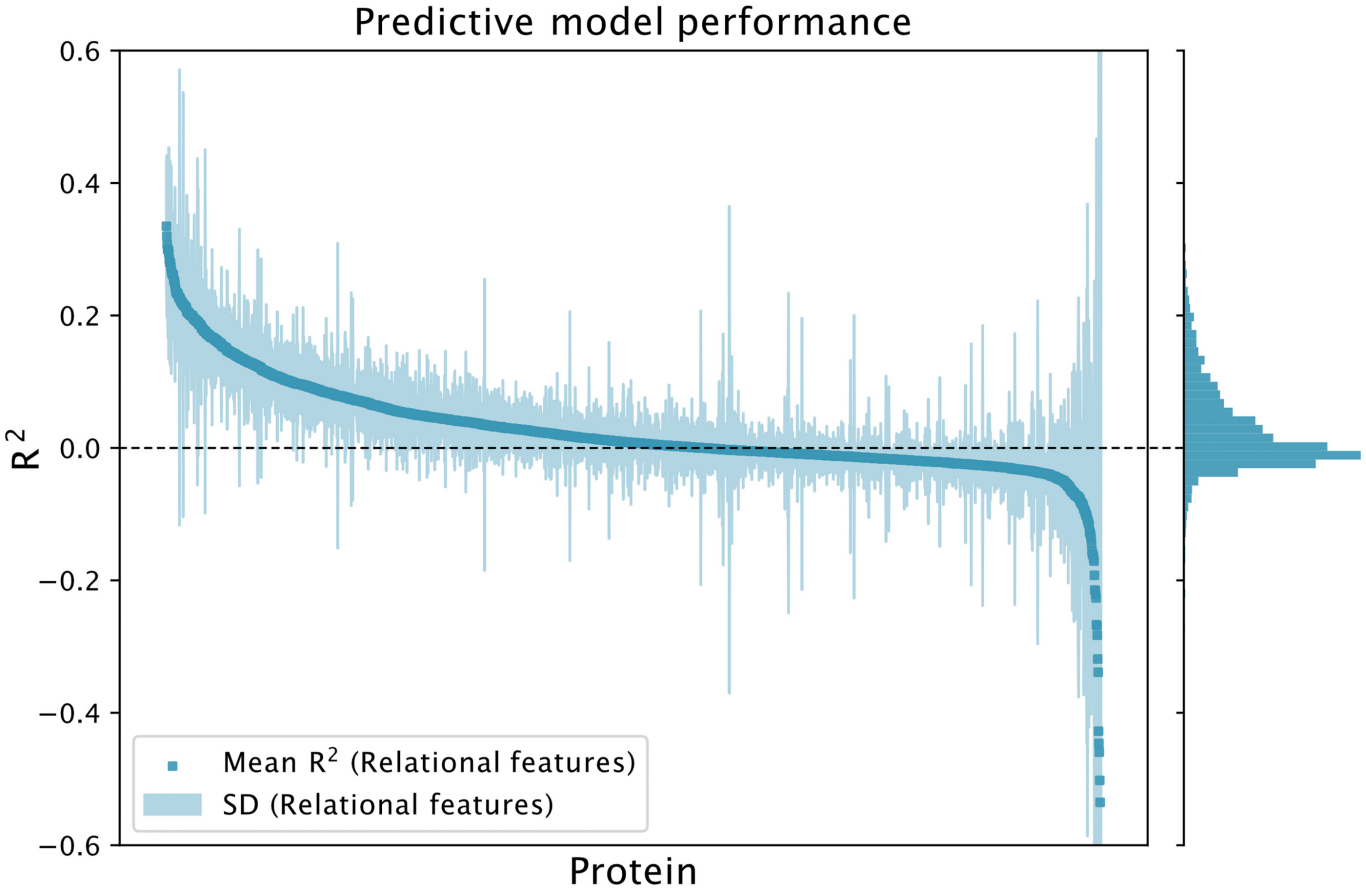
Protein abundance predictability from relational features according to mean $R^{2}$ across 2292 proteins in the dataset. The plot was truncated at $\pm 0.6\;R^{2}$ for visualization purposes. Each separate model was evaluated using 5-fold CV. One dot represents the mean score of a protein prediction model. The shaded area represents the standard deviation. The histogram denotes the distribution of mean $R^{2}$ for the predictive models.

As observed in Fig. 2A, protein abundance can be predicted from relational descriptors alone, with the majority of models showing a positive, although weak, coefficient of determination. A smaller subset of proteins show a stronger average predictive performance of above $0.3\;R^{2}$. This indicates that the coverage of relations present in the generated descriptors are sufficient to explain a significant fraction of the variation present in the abundances. The predictable fraction of proteins compared favourably to an explicit representation of the database, as measured by cross-validated $R^{2}$ (see Supplementary Notes SVII).

**Figure 3.**
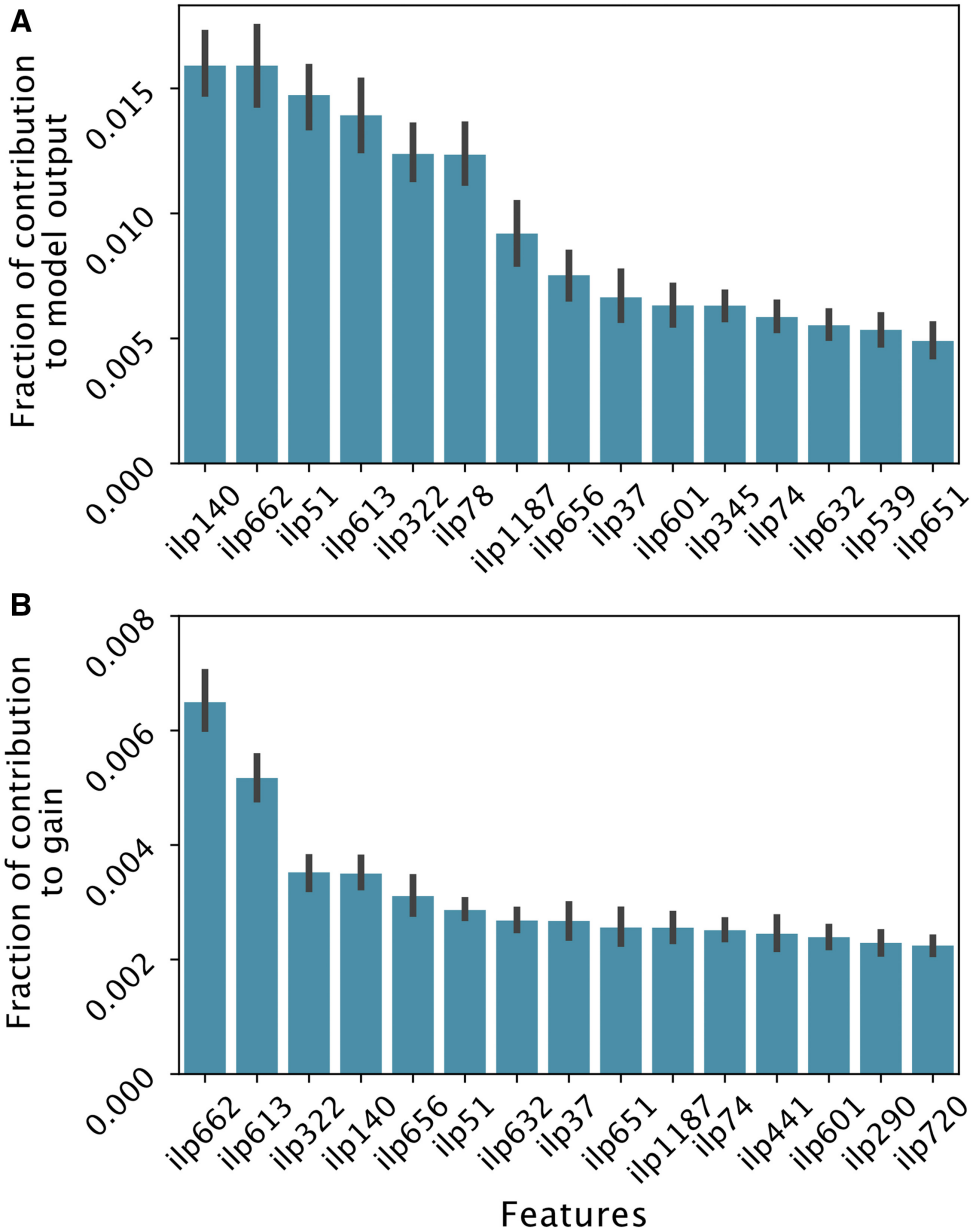
(A) Normalized mean(|SHAP|)-values of relational features across all available protein models with a positive coefficient of determination ($R^{2}>0$). (B) Normalized gain of relational features across all available protein models with a positive coefficient of determination ($R^{2}>0$). The error bars denote the 95% confidence interval. *ilp-* denotes that the feature is a generated descriptor. Explanations for these can be seen in Supplementary Notes SII.

As seen in Fig. 3A and B, features important for prediction (across the span of all available proteins) are descriptors with a large number of covered examples. Reasonably enough, abundance predictions were mainly dominated by logic programs (descriptions and English translations of these descriptors/programs can be seen in Supplementary Notes SII) containing sub-patterns with large effects on metabolism, including: terms regarding decreased fitness or growth defects and abnormal accumulations of α-amino acids (*ilp140, ilp662, ilp613, ilp322, ilp1187, ilp656, ilp601, ilp345, ilp632, ilp651*); exclusively abnormalities in α-amino acids (*ilp51, ilp78, ilp74, ilp441*); and irregularities in stress responses (*ilp539, ilp720*). *ilp-* denotes that the feature is a generated descriptor.

The five protein’s whose abundances that were shown to be the most predictable in these conditions are shown in Table 1. The majority of them (Ilv2, His4, Leu4, and Aat2) are involved in amino acid metabolism, and one being a component of the ATP synthase complex (Atp7). Among others, predictors of Ilv2 include markers of abnormalities in growth and accumulations of α-amino acids, such as valine and glutamine. The abundance of His4 seems even more strongly connected to amino acid accumulation, indicating abnormalities in serine and proline as important. Levels of Atp7—as its annotation would suggest—seems tied to malfunctions of respiratory growth, but also to general deviations in α-amino acid concentrations. Abundances of Leu4 implicated phenotypical markers such as growth irregulaties, decreased chronological lifespan and abnormal or decreased accumulations of valine and glutamine as important predictors. Lastly, predictions of Aat2 abundances denoted serine and proline as significant but also anomalies in respiratory growth and chronological lifespan. These observations corresponds well with literature, given that Ilv2, His4, Leu4, and Aat2 are enzymes directly involved with amino acid metabolism. Complete formulations of these descriptors (as seen in Table 1) can be found in Supplementary Notes SIII.

**Table 1.** The five protein models with the highest predictive performance (average $R^{2}$) using only generated relational descriptors, along with a short description and their primary predictive features.^a^

Name	$R^{2}$	SHAP^b^	Gain^c^
Ilv2 (Acetolactate synthase)	0.335	*ilp662*	*ilp1720*
His4 (Multifunctional)	0.320	*ilp443*	*ilp1790*
Atp7 (ATP Synthase 7)	0.307	*ilp1187*	*ilp1187*
Leu4 (2-isopropylmalate synthase)	0.300	*ilp601*	*ilp1720*
Aat2 (Aspartate aminotransferase)	0.299	*ilp1175*	*ilp1790*

a *ilp* denotes that the feature is a generated descriptor. Complete descriptions of these can be found in Supplementary Notes SIII.

b Highest ranked feature according to SHAP.

c Highest ranked feature according to gain.

As seen in Fig. 6A and B, closer inspection of the features predictive of Ilv2 abundance gives rise to a similar pattern as for previous observations, with features (descriptors) involving a large number of examples having high predictive power. These descriptors (see Supplementary Notes SIV) comprise of terms describing malfunctions of growth (*ilp662, ilp601*, and *ilp37*) or abnormalities in oxidative stress resistance coupled with accumulations of α-amino acids, specifically alanine (*ilp383* and *ilp972* respectively). Other descriptors only involved metabolite deviations, such as proline (*ilp441* and *ilp2032*), serine (*ilp1798*), valine (*ilp1798*), and lysine (*ilp2032*). The latter observations presumably due to the regulation of the protein primarily being under amino acid control, along with the fact that it catalyzes the first steps of isoleucine and valine synthesis (Falco *et al.* 1985, Xiao and Rank 1988). Inspection of the features showing maximum deviations of model output specify abnormalities in RNA accumulation [coupled with either activity in the mitochondria (*ilp1874*) or an increased accumulation of valine (*ilp1865*)] as highly affecting of specific predictions. In addition, it also identified several coupled statements, such as abnormal sporulation along with irregularities in methionine accumulation (*ilp1622*), metabolic proteins with biotinyl/lioypl domain profiles (*ilp1161*) and abnormal galactose utilization combined with aberrant tyrosine concentrations (*ilp1394*) as distinctly deviating model output.

### 3.3 Amino acids serves as predictors for protein abundance

The α-amino acid concentrations generated in Mülleder *et al.* (2016) were combined with a different propositionalized dataset (in which ORF-to-phenotype relations regarding α-amino acid accumulations had been removed from the Datalog-database prior to the pattern-search). Every protein prediction model now employed the same set of descriptors along with concentrations of nineteen different amino acids as predictive features. Overrepresentation-tests were performed for proteins with a stronger-than-normal connection to specific amino acids using ClusterProfiler (Wu *et al.* 2021). More in-depth descriptions of presented relational descriptors can be seen in Supplementary Notes SV.

**Figure 4.**
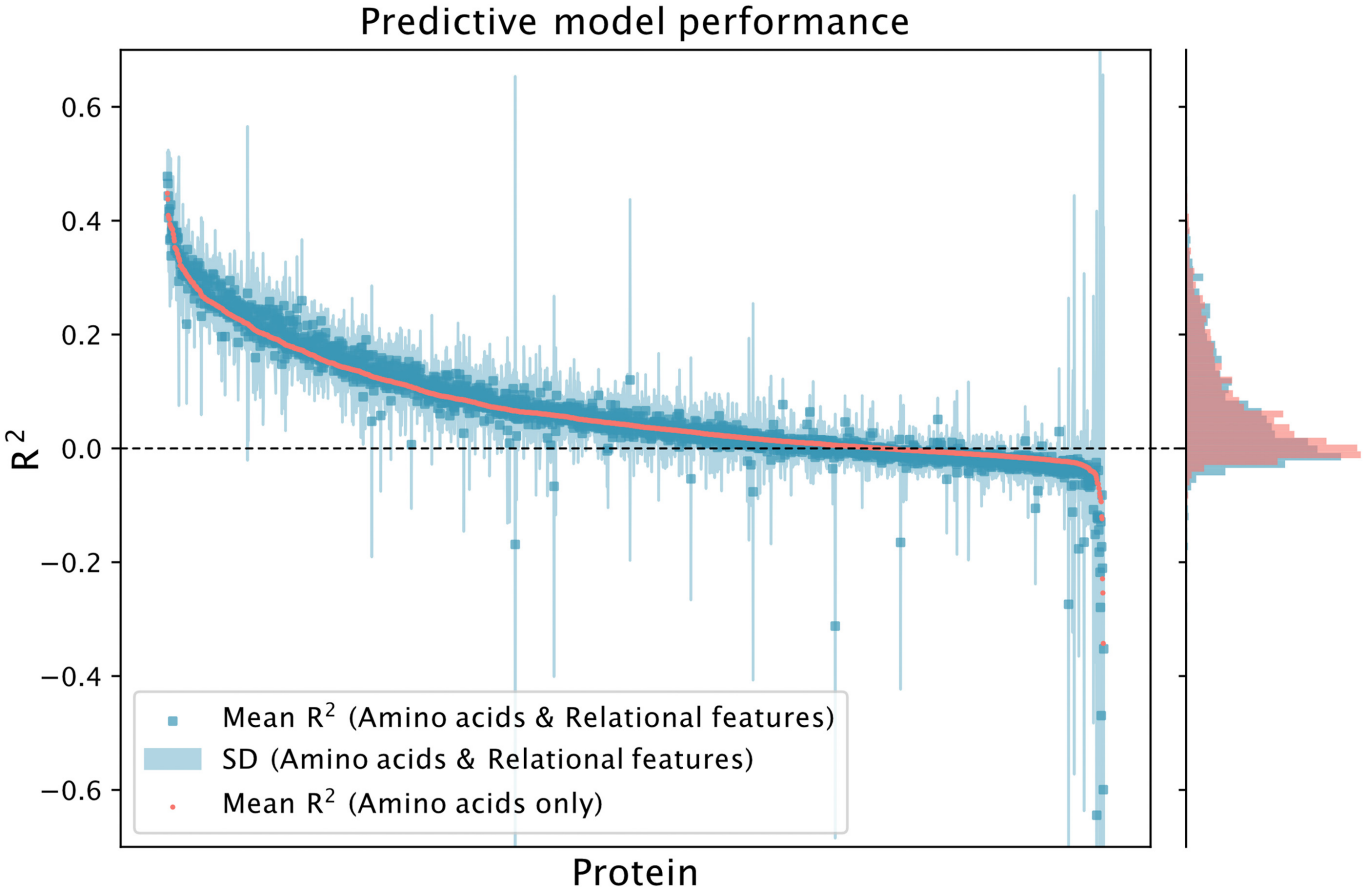
Protein abundance predictability from amino acid concentrations and relational features according to mean $R^{2}$ across 2292 proteins available in the dataset. The plot was truncated at $\pm 0.7\;R^{2}$ for visualization purposes. Each separate model was evaluated using 5-fold CV. One square represents the mean score of a protein prediction model using both amino acid concentrations and relational features, with the shaded area representing the standard deviation. The red dots represents the mean scores for predictive models using only amino acid concentrations as predictive features. The histogram denotes the distribution of mean $R^{2}$ for the predictive models.

As shown in Fig. 4, a large subset of protein abundances were predictable given matching concentration values of the nineteen α-amino acids, seemingly explaining as much as 44% of the variation in some cases (such as for Atp4 and Atp7). The majority of the protein models achieved meaningful, but low performance, with a significant portion of them barely outperforming the average value. By providing the predictive models with structured prior knowledge in the form of relational descriptors, improvements are seen in a portion of the proteins. These improvements were on top of the predictive models already achieving high performance using only concentration-based features, such as for the Atp4, Atp7, and His4.

**Figure 5.**
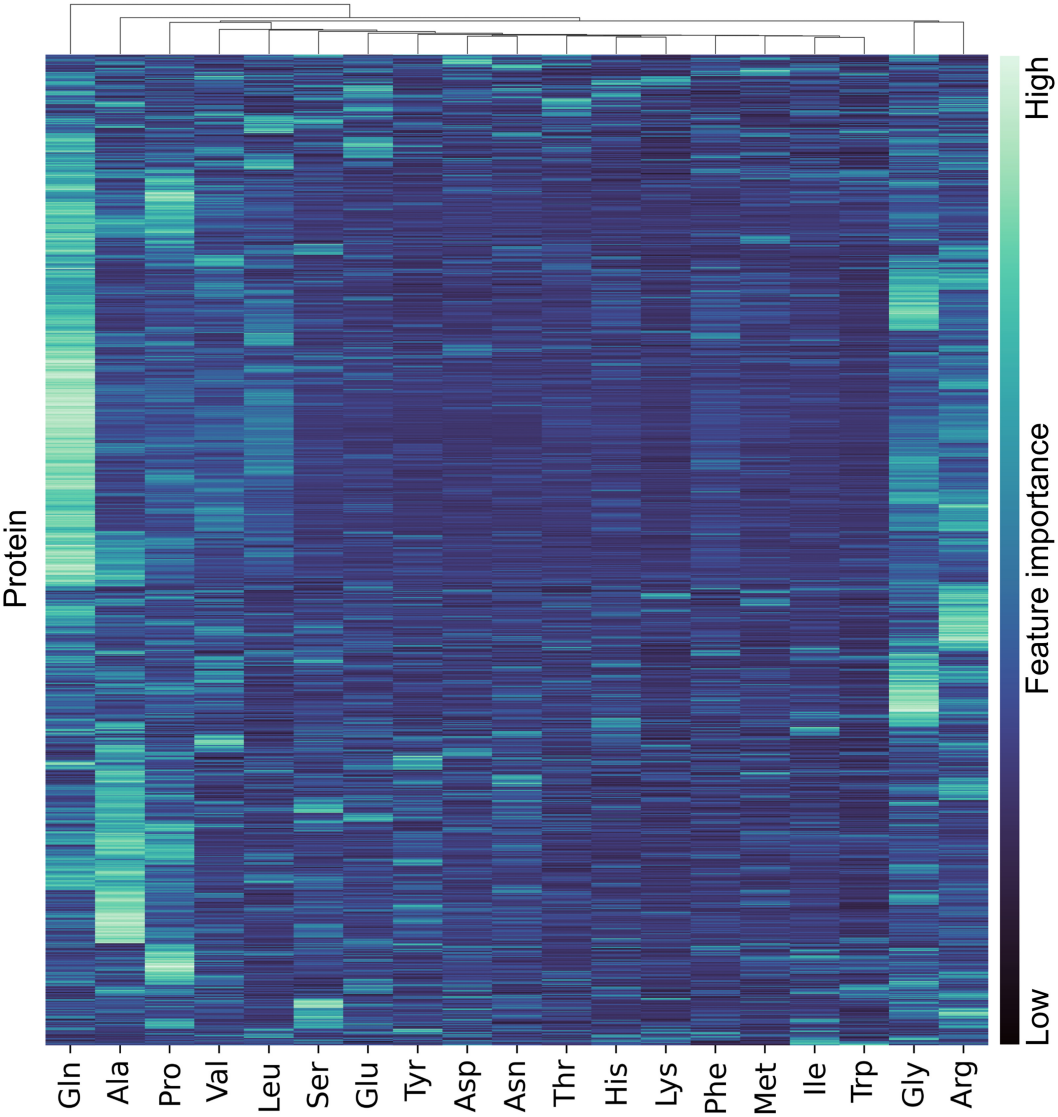
Heatmap of scaled amino acid feature importance values (SHAP) for all of the protein models with a positive coefficient of determination ($R^{2}>0$). Rows indicate the proteins that are being predicted (dependent variables), while columns indicate the predictive features (across the range of predictable proteins).

When looking for metabolites that provide the most significant effect on abundance across the whole space of predictable proteins, glutamine followed by alanine, glycine, arginine, and proline were over-represented (see Fig. 5). Glutamine seemed to have been one of the main predictors of global protein abundance in this particular setting. Overrepresentation tests for the proteins primarily predicted by glutamine (see Supplementary Fig. S5) show proteins that are connected to the metabolic process of small molecules, such as amino acids, purines, and the ncRNA (noncoding RNA) metabolic process. The proteins themselves mainly being active in the nucleolus or preribosomes. Proteins predicted (primarily) by glycine, showed a much more diverse set of overrepresentation-terms (see Supplementary Fig. S6), such as cytoplasmic translation, and terms regarding energy-generation (such as oxidative phosphorylation and the ATP metabolic process). Overrepresentation tests for proline-predicted proteins (see Supplementary Fig. S7) pointed towards cytosolic proteins, several protein-containing complexes, small molecule binding and biosynthetic processes for ribonucleic proteins, amides, peptides, and ribosomes.

**Figure 6.**
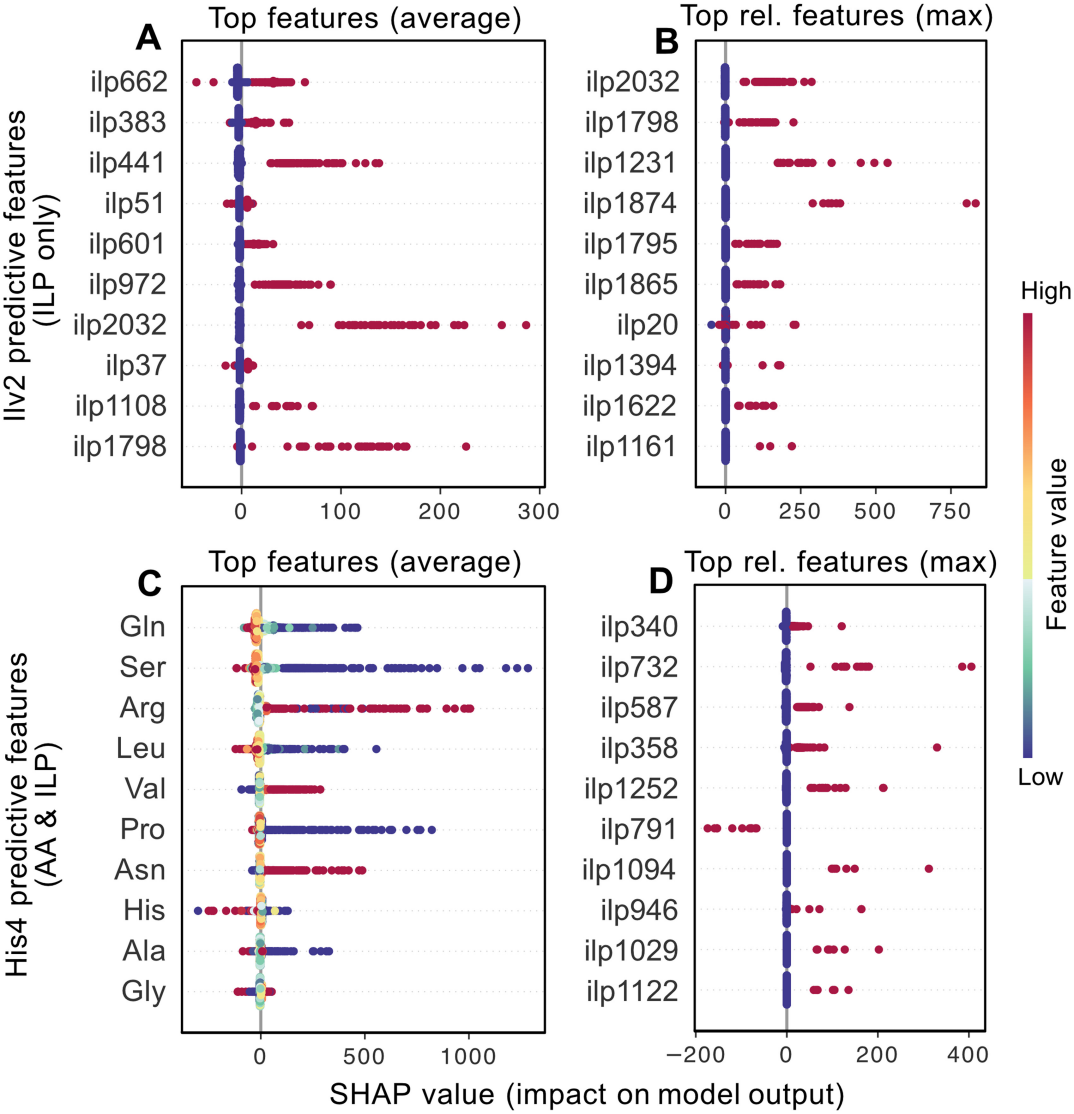
(A) Top features for the prediction of Ilv2, given only relational features. Sorted by average contribution in descending order. (B) Top relational features according to maximum change in model output for Ilv2, given only relational features. (C) Top features for the prediction of His4, given relational features and amino acid concentrations. Sorted by average contribution in descending order. (D) Top relational features according to maximum change in model output for His4, given relational features and amino acid concentrations. Each dot corresponds to one sample. *ilp-* denotes that the feature is a generated descriptor. Complete explanations for these descriptors can be seen in Supplementary Notes SIV and SV.

Closer investigation of His4 predictions (see Fig. 6C) demonstrate concentration-based features to be the main determinant of model output, with relational features deviating output in select cases. High concentrations of histidine lower the models output of His4 (HIStidine requiring 4) levels. Glutamine, serine, leucine, proline, and alanine all seem inversely related to His4 abundance, as low concentrations of these α-amino acids gave rise to higher abundances. The opposite behaviour was observed for arginine, valine, and asparagine. As can be seen in Fig. 6D and Supplementary Notes SV, there were some common patterns in the descriptors themselves, namely: Abnormalities in stress resistances (*ilp340, ilp587*, and *ilp358*); irregularities in RNA accumulations (*ilp1029*) or peptide distributions (*ilp791*); and nutrient utilization rates (*ilp732, ilp1252*, and *ilp1029*). The latter descriptors having identified groups of ORFs whose deletion causes increases or decreases in His-Leu utilization (a dipeptide formed from L-histidine and L-leucine residues). In addition, the intersection of abnormalities in telomere lengths and decreased $H^{+}$ accumulation (*ilp946*) were predictive of His4 abundance in a subset of samples, with the separate relations having close to no predictive power (see Supplementary Fig. S1).

## 4 Discussion

In this work, we leverage prior structured knowledge in yeast systems biology to construct a highly expressive Datalog database. This database contains information and relations that encompass, among others, gene-function, regulatory interactions, gene-to-metabolite relations, and phenotype observations (Cherry *et al.* 2012, Oughtred *et al.* 2021). We exploit this database by performing relational learning over its contents to generate binary data descriptors that we input directly into machine learning algorithms in the form of tabular data. This is done to produce more consistent and explainable predictions compared to more standard workflows, enabling us to more fully understand the regulation of protein abundances in *S.cerevisiae*. These descriptors are represented in the form of logic programs, which can be directly translated into English statements interpretable by domain experts, showcasing an advantage over popular methods such as deep neural networks (Orhobor *et al.* 2020). In this way we can make use of the advantages of attribute-value learners, such as XGBoost, to make efficient predictions while also identifying semantically meaningful factors that can be used to further explore yeast systems biology.

In contrast to recent biologically relevant deep-learning based methods, such as the work done in Ma *et al.* (2018), the prior knowledge can be represented as a propositional input, removing the need to specifically encode it in the structure (Fortelny and Bock 2020, Elmarakeby *et al.* 2021). This showcasing some advantage in flexibility and reduced complexity, but also allowing for easy addition of more, and additional types of priors. However—unlike the structure-based methods—the main disadvantage of the method used in this study is that it is a two-stage process. The relational descriptors that are extracted during frequent pattern mining are not necessarily useful in the predictive task—in this case the prediction of protein abundance.

In the worst case, this can lead to creating a high dimensionality problem with features that are equally or less informative than the baseline. This due to the search restricting access to a sufficient number of predictive features, as can be seen for the high variance, nonpredictable protein predictions in Figs 2 and 4. In some cases, it could also generate sparse and highly correlated features, which could lead to a decrease in predictive performance.

In this particular setting, the relational descriptors showed a favourable comparison (in terms of the coefficient of determination) to a comprehensive baseline representation (a binary relation matrix consisting of all relations present in the database) for the set of previously established predictable proteins (see Supplementary Notes SVII). This indicates that the pattern mining did return highly informative relational features for a large subset of proteins.

The proposed method itself also heavily relies on the completeness of the used theory, in this case being the functional and phenotypical annotations of the *S.cerevisiae* genome. Potential remedies, while still retaining some training efficiency, could be the use of computationally expensive feature selection techniques or even lifted methods such as LRNNs (Lifted Relational Neural Networks) (Šourek *et al.* 2021).

When interpreting the models via an explainable AI (XAI) framework, such as SHAP, caution needs to be taken as to not infer causality from these observations alone. In addition, the predictions themselves could be improved—due to the issues of generating over-fitting patterns, and highly correlated features confounding the predictions.

As seen in Fig. 2, the generated descriptors are capable of predicting the abundance to some degree, with globally important features mainly being phenotype-based (more specifically on biological accumulations of amino acids). While potentially an artifact of the data-collection itself—with data generated by the same lab—it still implies a meaningful connection between the amino acid concentrations and measured protein abundances (Mülleder *et al.* 2016, Messner *et al.* 2023). In addition, reasonably enough, abnormalities in growth are a strong predictor of overall protein abundance, not excluding the possibility that this could be an artifact of improper biomass normalization or other errors in the original dataset. It also signifies abnormalities in stress response (or the effect of stressors in general) as important for protein abundance distribution, especially in the context of oxidative stress—something that has been previously validated in literature for a subset of the proteins (Vogel *et al.* 2011).

More closely investigating the proteins with stronger connections to the generated descriptors generally indicated phenotype annotations as strong deciders of abundance. For example, the importance of valine (and in some cases isoleucine) as strong predictors of Ilv2 abundance, which is likely accurate given its name (IsoLeucine-plus-Valine-requiring 2) (Falco *et al.* 1985). SHAP-assisted feature interpretation also indicated inabilities to properly utilize galactose (usually paired with malfunctions in growth) as positive markers of Ilv2 abundance. The previous connection seems clearer given the fact that when *S.cerevisiae* is fed galactose instead of glucose as a main carbon source, the amino acid uptake is increased (as Ilv2 is under general amino acid control, and also synthesizes the first steps of valine and isoleucine biosynthesis) (Hothersall and Ahmed 2013). In addition, the model linked RNA-accumulations with Ilv2 abundance—likely due to the correlation between the intracellular levels of the two types of molecules—but more specifically, it linked the term with either mitochondrial activity or valine accumulations, resulting in vastly increased abundances for the covered examples (Vogel and Marcotte 2012, Lahtvee *et al.* 2017). This in turn likely pointing towards regulatory elements in the mitochondria involved in valine metabolism (outside of Ilv2), such as Bat1 (Takpho *et al.* 2018).

Additional investigation (follow up analysis) of the generated rules elucidated more detailed conditional dependencies between metabolites and proteomic abundances, with models being able to explain up to 47% of the observed variance. In addition, the models identified glutamine (followed by alanine, glycine, and proline) as a strong predictor of protein abundance. This possibly due to glutamines potential role as a replenisher of TCA (tricarboxylic acid cycle) intermediates, source of nitrogen, or through its implied role as a regulator of TORC1 (Durán *et al.* 2012, Jewell *et al.* 2015, Chantranupong and Sabatini 2016, Nilsson *et al.* 2020). Additionally, overrepresentation of glutamine-predicted proteins pointed towards metabolic processes of small molecules, the ribosome, and noncoding RNA. This result agrees with previous findings on epigenetic regulation in other eukaryotes (Lin *et al.* 2020).

Glycine involvement in protein abundance is connected with involvement in cytoplasmic translation and energy-generating processes such as ATP synthesis (more specifically the electron transport chain). Additionally proline, also a good indicator of protein abundance, is coupled to ribosome biogenesis and overall translation through its set of predicted proteins, something which is previously well known in literature (Melnikov *et al.* 2016).

By combining the two types of data (amino acid concentrations and relational features) we can achieve a better performing model. In case of specific proteins such as His4, the generated descriptors accurately catches sample-specific deviations in abundance. For example, the link between His4 levels and abnormalities in His-Leu uptake and mitochondrial metabolism. Or even patterns outside of the direct descriptive capacity of amino acids, such as telomere length and decreased intracellular $H^{+}$ accumulation. The latter potentially strengthening the positive association between histidine and aging-effecting stressors (Canfield and Bradshaw 2019). Both structural deviations in telomeres and low intracellular pH are previously known to cause inconsistencies in replicative aging in eukaryotes, with the model indicating that the intersection of the two could be linked with an increase of His4 levels (Yoshida *et al.* 2010, Eigenfeld *et al.* 2021).

Additionally, the proposed methodology also partially verifies existing literature by predictively connecting qualitative biological concepts, derived through years of work on yeast systems biology, to quantified abundances of biomolecules. While the predictive power of these models are imperfect, they showcase the relative completeness of the current functional and phenotypical annotations for *S.cerevisiae*. Machine learning methods integrating domain-knowledge in an interpretable way could be used to understand abundances of various biomolecules through connections to completely different types of data. The models (and their interpretation) could also be used to identify protein function.

## 5 Conclusion

Integrating domain knowledge from curated biological databases into our machine learning models through logic programs can significantly improve their interpretability and performance.

Interpretable models enable us to better understand regulatory rules regarding protein abundance in *S.cerevisiae*.

## Supplementary Material

btae050_Supplementary_Data

## Data Availability

All code, data, and generated features are accessible at https://github.com/DanielBrunnsaker/ProtPredict.
