# Supplementary material for: Interpreting protein abundance in Saccharomyces cerevisiae through relational learning
Source: Bioinformatics. 2024 Jan 25;40(2):btae050. doi: 10.1093/bioinformatics/btae050 (PMC10868306; doi:10.1093/bioinformatics/btae050)
Supplement: btae050_Supplementary_Data [file btae050_supplementary_data.pdf]

SUPPLEMENTARY MATERIAL FOR INTERPRETING PROTEIN ABUNDANCE IN *SACCHAROMYCES CEREVISIAE* THROUGH RELATIONAL LEARNING

Daniel Brunnsåker[1], Filip Kronström[1], Ievgeniia A. Tiukova[2,3] and Ross D. King[1,4,5]

[1]Department of Computer Science and Engineering, Chalmers University of Technology, Göteborg, Sweden.
[2]Department of Life Sciences, Chalmers University of Technology, Göteborg, Sweden.
[3]Division of Industrial Biotechnology, KTH Royal Institute of Technology, Stockholm, Sweden.
[4]Department of Chemical Engineering and Biotechnology, University of Cambridge, Cambridge, United Kingdom.
[5]Alan Turing Institute, London, United Kingdom.

Contents

1 Supplementary Notes I **3**

2 Supplementary Notes II **5**

2.1 Relational descriptors for Figure 2AB 5

3 Supplementary Notes III **8**

3.1 Relational descriptors for Table 1 8

4 Supplementary Notes IV **9**

4.1 Relational descriptors for Figure6AB 9

5 Supplementary Notes V **12**

5.1 Relational descriptors for Figure6CD 12

5.2 Descriptor comparison, His4 13

6 Supplementary Notes VI (Benchmarking) **15**

7 Supplementary Notes VII (Representation comparison) **16**

8 Supplementary Notes VIII (Over-representation tests) **17**

1 Supplementary Notes I

Allowed relations in the frequent pattern search

Below are the allowed relations (modes) in the frequent pattern search, along with a summary of the knowledge they encode. +gene and -gene denotes the input and output variables respectively, in this case the open reading frames corresponding to the deletions/deletant strain. Note that "gene" and "open reading frame" are used interchangeably in the context of this frequent pattern search.

$$\mathsf{gene\_metabolite}(+\mathsf{gene},\ \#\mathsf{metabolite}) \tag{1}$$

Gene-to-metabolite relations were extracted from the gene-metabolite graph in Yeast8[1]. It implies that the gene (+gene) codes for a protein (or that the protein is part of a complex) that catalyzes a reaction involving a specific metabolite (#metabolite).

$$\mathsf{gene\_pathway}(+\mathsf{gene},\ \#\mathsf{pathway}) \tag{2}$$

This relation points towards a genes/proteins (+gene) involvement in a biological pathway (#pathway). These were downloaded from YeastMine[2].

$$\mathsf{gene\_chromosome}(+\mathsf{gene},\ \#\mathsf{chromosome}) \tag{3}$$

This relation indicates the chromosome (#chromosome) on which the gene (+gene) is located. These were downloaded from YeastMine[2].

$$\mathsf{gene\_nullphenotype}(+\mathsf{gene},\ \#\mathsf{phenotype}) \tag{4}$$

Gene-to-(null)phenotype relations indicate the observed phenotype (#phenotype) caused by the deletion of the gene (+gene). Dose-dependent phenotypes, such as toxin resistance and resistance to chemicals were removed from the search. These relations were downloaded from YeastMine[2].

$$\mathsf{gene\_nullphenotype\_chemical}(+\mathsf{gene},\ \#\mathsf{phenotype},\ \#\mathsf{chemical}) \tag{5}$$

Gene-to-(null)phenotype relations (of arity 3) that indicate the observed phenotype (#phenotype) connected to specific chemical (#chemical) abundances, caused by either the deletion of the gene (+gene) or a condition specified by the chemical (#chemical). These relations were downloaded from YeastMine[2].

$$\mathsf{gene\_hasproteindomain}(+\mathsf{gene},\ \#\mathsf{domain}) \tag{6}$$

Relation indicating links between a specific protein (+gene) and its identified domains (#domain) (such as a zinc-finger domain). These relations were downloaded from YeastMine[2].

$$\text{involved\_in}(+\text{gene},\ \#\text{function}) \tag{7}$$
$$\text{enables}(+\text{gene},\ \#\text{function}) \tag{8}$$
$$\text{part\_of}(+\text{gene},\ \#\text{function}) \tag{9}$$
$$\text{contributes\_to}(+\text{gene},\ \#\text{function}) \tag{10}$$
$$\text{colocalizes\_with}(+\text{gene},\ \#\text{function}) \tag{11}$$
$$\text{acts\_upstream\_of}(+\text{gene},\ \#\text{function}) \tag{12}$$
$$\text{acts\_upstream\_of\_negative\_effect}(+\text{gene},\ \#\text{function}) \tag{13}$$
$$\text{acts\_upstream\_of\_positive\_effect}(+\text{gene},\ \#\text{function}) \tag{14}$$
$$\text{acts\_upstream\_of\_or\_within\_positive\_effect}(+\text{gene},\ \#\text{function}) \tag{15}$$
$$\text{is\_active\_in}(+\text{gene},\ \#\text{function}) \tag{16}$$
$$\text{located\_in}(+\text{gene},\ \#\text{function}) \tag{17}$$
$$\tag{18}$$

Relations mirroring the Gene Ontology[3]. These relations were downloaded from YeastMine[2].
For more information, see `https://geneontology.org/docs/ontology-relations/`.

$$\text{enzymatic\_interaction}(+\text{gene},\ -\text{gene}) \tag{19}$$

These are extracted from the gene-gene graph in Yeast8[1], a relation would imply that the two genes (+gene/-gene) code for proteins that catalyze a reaction containing a shared metabolite.

$$\text{regulates}(+\text{gene}, -\text{gene},\ \#\text{type}) \tag{20}$$

The relation describes a regulatory interaction between two proteins/genes (+gene regulates -gene), and the regulatory type (#type) of its interaction (described by the type of protein that +gene is, e.g. a transcription factor).

$$\text{regulateBy}(+\text{gene}, -\text{gene},\ \#\text{type}) \tag{21}$$

Inverse to the relation above. It describes a regulatory interaction in which a gene/protein (+gene) is regulated by a protein (-gene), with the type (#type) of interaction encoded by the type of protein that -gene is.

2 Supplementary Notes II

2.1 Relational descriptors for Figure 2AB

(**ilp140**) ORF(A) :=

ORF_nullPhenotype(A, Abnormal competitive fitness),

ORF_nullPhenotypeChemical(A, Increased chemical compound accumulation, Glycine) [101]

ORFs(A) whose deletion causes abnormal competitive fitness and increased accumulations of glycine (n = 101).

(**ilp662**) ORF(A) :=

ORF_nullPhenotype(A, Decreased rate vegetative growth),

ORF_nullPhenotypeChemical(A, Abnormal chemical compound accumulation, $\alpha -$ amino acid) [644]

ORFs(A) whose deletion causes a decreased rate of vegetative growth and abnormal accumulations of α-amino acids (n = 644).

(**ilp51**) ORF(A) :=

ORF_nullPhenotypeChemical(A, Abnormal chemical compound accumulation, $\alpha -$ amino acid) [1459]

ORFs(A) whose deletion causes abnormal accumulations of α-amino acids (n = 1459).

(**ilp613**) ORF(A) :=

ORF_nullPhenotype(A, Abnormal rate vegetative growth),

ORF_nullPhenotypeChemical(A, Abnormal chemical compound accumulation, $\alpha -$ amino acid) [647]

ORFs(A) whose deletion causes an abnormal rate of vegetative growth and abnormal accumulations of α-amino acids (n = 647).

(**ilp322**) ORF(A) :=

ORF_nullPhenotype(A, Decreased competitive fitness),

ORF_nullPhenotypeChemical(A, Abnormal chemical compound accumulation, $\alpha -$ amino acid) [1225]

ORFs(A) whose deletion causes decreased competitive fitness and abnormal accumulations of α-amino acids (n = 1225).

(**ilp78**) ORF(A) :=

ORF_nullPhenotypeChemical(A, Increased chemical compound accumulation, Valine) [188]

ORFs(A) whose deletion causes increased accumulations of valine (n = 188).

(**ilp1187**) ORF(A) :=

ORF_nullPhenotype(A, Absent respiratory growth),

ORF_nullPhenotypeChemical(A, Abnormal chemical compound accumulation, $\alpha -$ amino acid) [321]

ORFs(A) whose deletion causes an absence of respiratory growth and abnormal accumulations of α-amino acids (n

= 321).

(**ilp656**) ORF(A) :=

ORF_nullPhenotype(A, Decreased rate vegetative growth),

ORF_nullPhenotype(A, Increased heat sensitivity) [644]

ORFs(A) whose deletion causes a decreased rate of vegetative growth and increased heat sensitivity (n = 644).

(**ilp37**) ORF(A) :=

ORF_nullPhenotype((A, Decreased rate vegetative growth) [1106]

ORFs(A) whose deletion causes a decreased rate of vegetative growth (n = 1106).

(**ilp601**) ORF(A) :=

ORF_nullPhenotype(A, Abnormal rate vegetative growth),

ORF_nullPhenotype(A, Decreased chronological lifespan) [474]

ORFs(A) whose deletion causes an abnormal rate of vegetative growth and a decreased chronological lifespan (n = 474).

(**ilp345**) ORF(A) :=

ORF_nullPhenotype(A, Decreased competitive fitness),

ORF_nullPhenotypeChemical(A, Increased chemical compound accumulation, Glycine) [87]

ORFs(A) whose deletion causes decreased competitive fitness and an increased accumulation of glycine (n = 87).

(**ilp74**) ORF(A) :=

ORF_nullPhenotypeChemical(A, Increased chemical compound accumulation, Glycine) [105]

ORFs(A) whose deletion causes increased accumulations of glycine (n = 105).

(**ilp632**) ORF(A) :=

ORF_nullPhenotype(A, Abnormal rate vegetative growth),

ORF_nullPhenotypeChemical(A, Decreased chemical compound accumulation, Proline) [82]

ORFs(A) whose deletion causes an abnormal rate of vegetative growth and a decreased accumulation of proline (n = 82).

(**ilp539**) ORF(A) :=

ORF_nullPhenotype(A, Abnormal stress resistance),

ORF_nullPhenotype(A, Increased oxidative stress resistance) [421]

ORFs(A) whose deletion causes overall abnormalities in stress resistance and an increased oxidative stress resistance

(n = 421).

(**ilp651**) ORF(A) :=

ORF_nullPhenotype(A, Decreased rate vegetative growth),

ORF_nullPhenotype(A, Decreased competitive fitness) [1019]

ORFs(A) whose deletion causes a decreased rate of vegetative growth and decreased competitive fitness (n = 1019).

(**ilp441**) ORF(A) :=

ORF_nullPhenotypeChemical(A, Abnormal chemical compound accumulation, $\alpha -$ amino acid),

ORF_nullPhenotypeChemical(A, Decreased chemical compound accumulation, Proline) [121]

ORFs(A) whose deletion causes abnormal overall accumulations of α-amino acids and decreased accumulations of proline (n = 121).

(**ilp290**) ORF(A) :=

RegulatedBy(A, B, Transcription factor),

ORF_nullPhenotypeChemical(A, Decreased chemical compound accumulation, Proline) [110]

ORFs(A) whose deletion causes a decreased accumulation of proline, and are regulated by a transcription factor (B) (n = 110).

(**ilp720**) ORF(A) :=

ORF_nullPhenotype(A, Abnormal heat sensitivity),

ORF_nullPhenotypeChemical(A, Abnormal chemical compound accumulation, $\alpha -$ amino acid) [633]

ORFs(A) whose deletion causes abnormalities in heat sensitivity and abnormal accumulations of α-amino acids (n = 633).

3 Supplementary Notes III

3.1 Relational descriptors for Table 1

(**ilp662**) ORF(A) :=

ORF_nullPhenotype(A, Decreased rate vegetative growth),

ORF_nullPhenotypeChemical(A, Increased chemical compound accumulation, $\alpha -$ amino acid) [644]

ORFs(A) whose deletion causes a decreased rate of vegetative growth and increased accumulations of α-amino acids (n = 644).

(**ilp1720**) ORF(A) :=

ORF_nullPhenotypeChemical(A, Abnormal chemical compound accumulation, Valine),

ORF_nullPhenotypeChemical(A, Decreased chemical compound accumulation, Glutamine) [27]

ORFs(A) whose deletion causes abnormal accumulations of valine and decreased accumulations of glutamine (n = 27).

(**ilp443**) ORF(A) :=

ORF_nullPhenotypeChemical(A, Abnormal chemical compound accumulation, $\alpha -$ amino acid),

ORF_nullPhenotypeChemical(A, Decreased chemical compound accumulation, Serine) [134]

ORFs(A) whose deletion causes abnormal accumulations of α-amino acids and decreased accumulations of serine (n = 134).

(**ilp1790**) ORF(A) :=

ORF_nullPhenotypeChemical(A, Abnormal chemical compound accumulation, Serine),

ORF_nullPhenotypeChemical(A, Decreased chemical compound accumulation, Proline) [40]

ORFs(A) whose deletion causes abnormal accumulations of serine and decreased accumulations of proline (n = 40).

(**ilp1187**) ORF(A) :=

ORF_nullPhenotype(A, Absent respiratory growth),

ORF_nullPhenotypeChemical(A, Abnormal chemical compound accumulation, $\alpha -$ amino acid) [321]

ORFs(A) whose deletion causes an absence of respiratory growth and abnormal accumulations of α-amino acids (n = 321).

(**ilp601**) ORF(A) :=

ORF_nullPhenotype(A, Abnormal rate vegetative growth),

ORF_nullPhenotype(A, Decreased chronological lifespan) [474]

ORFs(A) whose deletion causes an abnormal rate of vegetative growth and a decreased chronological lifespan (n =

474).

(**ilp1175**) ORF(A) :=

ORF_nullPhenotype(A, Absent respiratory growth),

ORF_nullPhenotype(A, Decreased chronological lifespan) [327]

ORFs(A) whose deletion causes an absence of respiratory growth and decreased chronological lifespan (n = 327).

4 Supplementary Notes IV

4.1 Relational descriptors for Figure6AB

(**ilp662**) ORF(A) :=

ORF_nullPhenotype(A, Decreased rate vegetative growth),

ORF_nullPhenotypeChemical(A, Abnormal chemical compound accumulation, $\alpha -$ amino acid) [644]

ORFs(A) whose deletion causes a decreased rate of vegetative growth and abnormal accumulations of α-amino acids (n = 644).

(**ilp383**) ORF(A) :=

ORF_nullPhenotype(A, Abnormal oxidative stress resistance),

ORF_nullPhenotypeChemical(A, Abnormal chemical compound accumulation, $\alpha -$ amino acid) [751]

ORFs(A) whose deletion causes an abnormal oxidative stress resistance and abnormal accumulations of α-amino acids (n = 751).

(**ilp441**) ORF(A) :=

ORF_nullPhenotypeChemical(A, Abnormal chemical compound accumulation, $\alpha -$ amino acid),

ORF_nullPhenotypeChemical(A, Decreased chemical compound accumulation, proline) [121]

ORFs(A) whose deletion causes abnormal accumulations of α-amino acids and decreased accumulations of proline (n = 121).

(**ilp51**) ORF(A) :=

ORF_nullPhenotypeChemical(A, Abnormal chemical compound accumulation, $\alpha -$ amino acid) [1459]

ORFs(A) whose deletion causes abnormal accumulations of α-amino acids (n = 1459).

(**ilp601**) ORF(A) :=

ORF_nullPhenotype(A, Abnormal rate vegetative growth),

ORF_nullPhenotype(A, Decreased chronological lifespan) [474]

ORFs(A) whose deletion causes an abnormal rate of vegetative growth and a decrease in chronological lifespan (n =

474).

(**ilp972**) ORF(A) :=

ORF_nullPhenotype(A, Increased oxidative stress resistance),

ORF_nullPhenotypeChemical(A, Abnormal chemical compound accumulation, alanine) [137]

ORFs(A) whose deletion causes increased oxidative stress resistance and abnormal accumulations of alanine (n = 137).

(**ilp2032**) ORF(A) :=

ORF_nullPhenotypeChemical(A, Abnormal chemical compound accumulation, lysine),

ORF_nullPhenotypeChemical(A, Decreased chemical compound accumulation, proline) [38]

ORFs(A) whose deletion causes abnormal accumulations of lysine and decreased accumulations of proline (n = 38).

(**ilp37**) ORF(A) :=

ORF_nullPhenotype(A, Decreased rate vegetative growth) [1106]

ORFs(A) whose deletion causes a decrease in rate of vegetative growth (n = 1106).

(**ilp1108**) ORF(A) :=

EnzymaticInteraction(A, A), ORF_nullPhenotype(A, Abnormal endoplasmic reticulum morphology) [20]

Metabolic genes/ORFs(A) whose deletion causes an abnormal endoplasmic reticulum morphology (n = 20).

(**ilp1798**) ORF(A) :=

ORF_nullPhenotypeChemical(A, Abnormal chemical compound accumulation, serine),

ORF_nullPhenotypeChemical(A, Increased chemical compound accumulation, valine) [38]

ORFs(A) whose deletion causes abnormal accumulations of serine and increased accumulations of valine (n = 38).

(**ilp1231**) ORF(A) :=

ORF_nullPhenotype(A, Decreased rate respiratory growth),

ORF_nullPhenotype(A, Decreased nutrient utilization) [21]

ORFs(A) whose deletion causes an abnormal rate of vegetative growth and a decrease in chronological lifespan (n = 21).

(**ilp1874**) ORF(A) :=

ORF_nullPhenotype(A, Abnormal RNA accumulation),

IsActiveIn(A, Mitochondrion) [9]

ORFs(A) whose deletion causes abnormal RNA accumulation, and is active in the mitochondrion (n = 9).

(**ilp1795**) ORF(A) :=

ORF_nullPhenotypeChemical(A, Abnormal chemical compound accumulation, serine),

ORF_nullPhenotypeChemical(A, Increased chemical compound accumulation, isoleucine) [22]

ORFs(A) whose deletion causes abnormal accumulations of serine and increased accumulations of isoleucine (n = 22).

(**ilp1865**) ORF(A) :=

ORF_nullPhenotype(A, Abnormal RNA accumulation),

ORF_nullPhenotypeChemical(A, Increased chemical compound accumulation, valine) [17]

ORFs(A) whose deletion causes abnormal accumulations of RNA and increased accumulations of valine (n = 17).

(**ilp20**) ORF(A) :=

ORF_nullPhenotype(A, Abnormal rate respiratory growth) [696]

ORFs(A) whose deletion causes abnormalities in respiratory growth rates (n = 696).

(**ilp1394**) ORF(A) :=

ORF_nullPhenotypeChemical(A, Abnormal rate utilization of carbon source, galactose),

ORF_nullPhenotypeChemical(A, Abnormal chemical compound accumulation, tyrosine) [53]

ORFs(A) whose deletion causes abnormalities in utilization rates of galactose and causes increased accumulations of tyrosine (n = 53).

(**ilp1622**) ORF(A) :=

ORF_nullPhenotype(A, Abnormal sporulation),

ORF_nullPhenotypeChemical(A, Abnormal chemical compound accumulation, methionine) [10]

ORFs(A) whose deletion causes abnormal sporulation and abnormal accumulations of methionine (n = 10).

(**ilp1161**) ORF(A) :=

EnzymaticInteraction(A, A), ORF_HasProteinDomain(A, Biotinyl/lipoyl domain profile) [4]

Metabolic genes/ORFs(A) that code for a protein that has a biotinyl/lipoyl domain profile (n = 4).

5 Supplementary Notes V

5.1 Relational descriptors for Figure6CD

(**ilp340**) ORF(A) :=

ORF_nullPhenotype(A, Abnormal stress resistance),

ORF_nullPhenotype(A, Increased oxidative stress resistance) [421]

ORFs(A) whose deletion causes an abnormal stress resistance, and an increased oxidative stress resistance (n = 421).

(**ilp732**) ORF(A) :=

ORF_nullPhenotype(A, Decreased rate respiratory growth),

ORF_nullPhenotype(A, Decreased nutrient utilization) [21]

ORFs(A) whose deletion causes a decrease of rate in respiratory growth, and a decreased nutrient utilization (n = 21).

(**ilp587**) ORF(A) :=

ORF_nullPhenotype(A, Increased oxidative stress resistance), IsActiveIn(A, Cytoplasm) [82]

ORFs(A) whose deletion causes an increase in oxidative stress resistance, and are active in the cytoplasm (n = 82).

(**ilp358**) ORF(A) :=

ORF_nullPhenotype(A, Abnormal stress resistance), IsActiveIn(A, Cytoplasm) [132]

ORFs(A) whose deletion causes an abnormal oxidative stress resistance, and are active in the cytoplasm (n = 132).

(**ilp1252**) ORF(A) :=

ORF_nullPhenotype(A, Decreased nutrient utilization), PartOf(A, Mitochondrion) [19]

ORFs(A) whose deletion causes a decreased nutrient utilization, and are part of the mitochondrion (n = 19).

(**ilp791**) ORF(A) :=

ORF_nullPhenotype(A, Abnormal protein/peptide distribution),

ORF_nullPhenotypeChemical(A, Abnormal utilization rate of carbon source, glucose) [14]

ORFs(A) whose deletion causes an abnormal protein/peptide distribution, and an abnormal utilization rate of glucose (n = 14).

(**ilp1094**) ORF(A) :=

ORF_nullPhenotype(A, Abnormal nutrient utilization), IsActiveIn(A, Mitochondrion) [9]

ORFs(A) whose deletion causes an abnormal nutrient utilization, and are active in the mitochondrion (n = 9).

(**ilp946**) ORF(A) :=

ORF_nullPhenotype(A, Abnormal telomere length),

ORF_nullPhenotypeChemical(A, Decreased chemical compound accumulation, proton) [13]

ORFs(A) whose deletion causes an abnormal telomere length, and cause a decreased accumulation of protons (n = 13).

(**ilp1029**) ORF(A) :=

ORF_nullPhenotype(A, Abnormal endoplasmic reticulum morphology),

ORF_nullPhenotype(A, Increased RNA accumulation) [9]

ORFs(A) whose deletion causes an abnormal endoplasmic reticulum morphology, and cause an increased accumulation of RNA (n = 9).

(**ilp1029**) ORF(A) :=

ORF_Metabolite(A, NAD), ORF_nullPhenotype(A, Abnormal nutrient utilization) [6]

ORFs(A) whose deletion causes an abnormal nutrient utilization, and express an enzyme which interacts with NAD (n = 6).

5.2 Descriptor comparison, His4

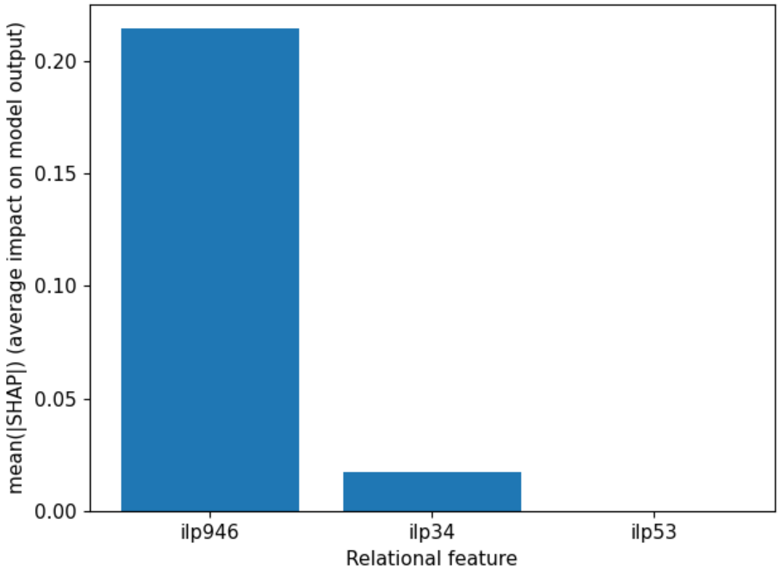

Figure 1: Barplot showing the increase in predictive power (impact on model output) for His4 abundance for the full relational descriptor (*ilp946*) when compared to its subrelations (*ilp34* and *ilp53*).

(**ilp946**) ORF(A) :=

ORF_nullPhenotype(A, Abnormal telomere length),

ORF_nullPhenotypeChemical(A, Decreased chemical compound accumulation, proton) [13]

ORFs(A) whose deletion causes an abnormal telomere length, and cause a decreased accumulation of protons (n =

13).

(**ilp34**) ORF(A) :=

ORF_nullPhenotype(A, Abnormal telomere length) [241]

ORFs(A) whose deletion causes an abnormal telomere length (n = 241).

(**ilp53**) ORF(A) :=

ORF_nullPhenotypeChemical(A, Decreased chemical compound accumulation, proton) [100]

ORFs(A) whose deletion causes a decreased accumulation of protons (n = 100).

6 Supplementary Notes VI (Benchmarking)

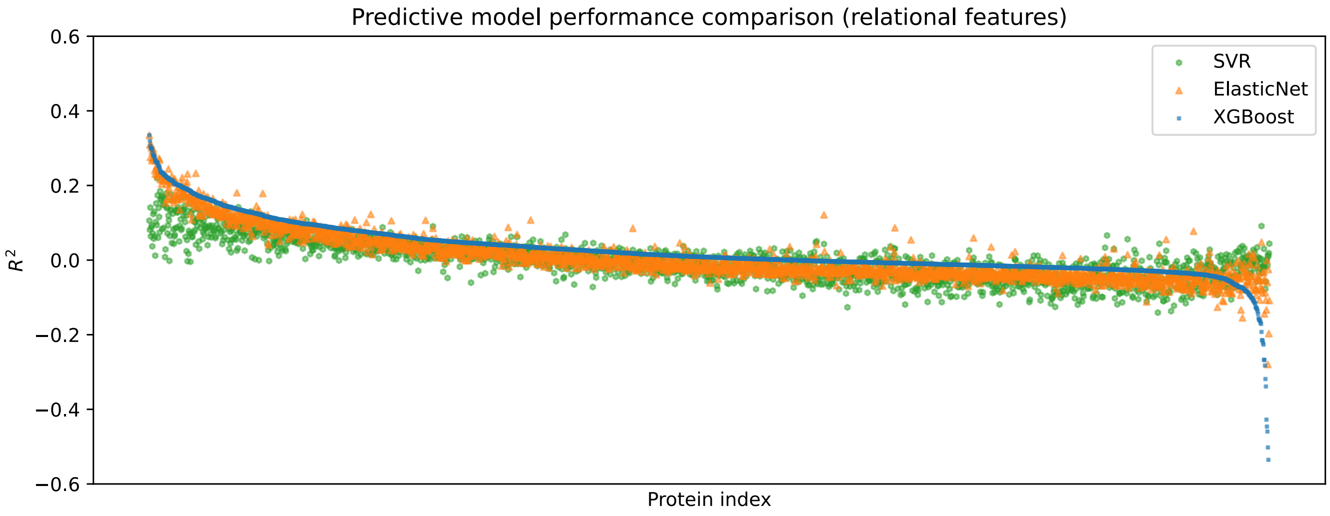

Figure 2: Benchmarking results for three supervised learning algorithms (XGBoost, ElasticNet and support vector regression (SVR)) using relational features only. Visualized is protein abundance predictability according to mean R^2 across 2292 proteins in the data-set. Sorted in descending order according to mean R^2 for XGBoost. The plot was truncated at ± 0.6 R^2 for visualization purposes. Each separate model was evaluated using 5-fold CV. One marker represents the mean score for one protein using different supervised learning algorithms. The hyper-parameters for each model were tuned on the first example protein in the data-set (A5Z2X5/Min8/YPR010C-A) with cross-validated bayesian optimization, using sci-kit optimize (v0.9.0)

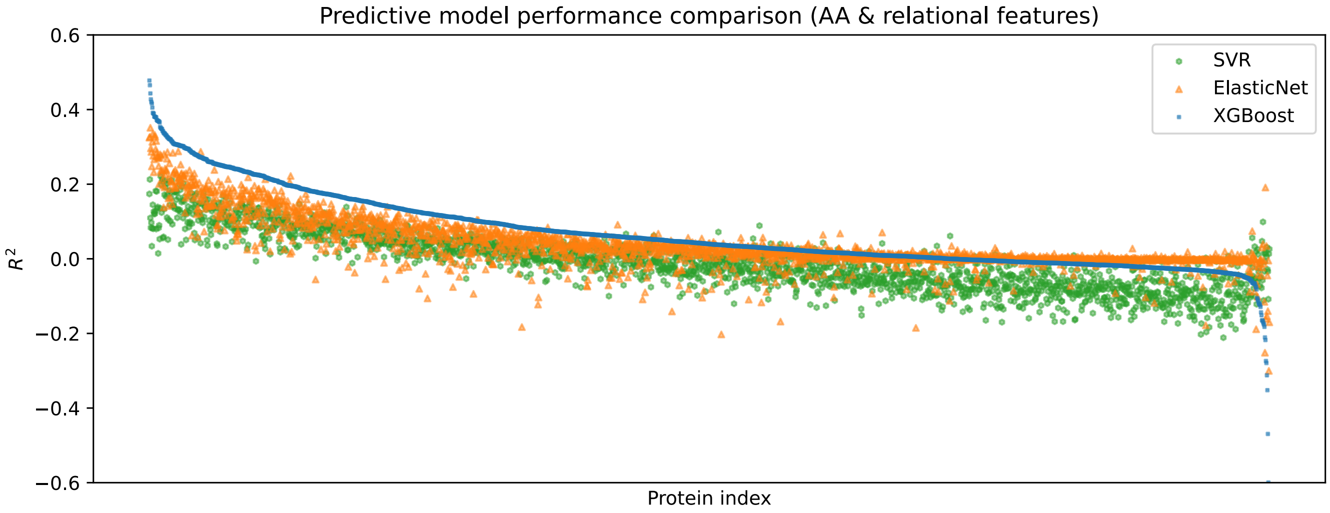

Figure 3: Benchmarking results for three supervised learning algorithms (XGBoost, ElasticNet and support vector regression (SVR)) using relational features and amino acid concentrations. Visualized is protein abundance predictability according to mean R^2 across 2292 proteins in the data-set. Sorted in descending order according to mean R^2 for XGBoost. The plot was truncated at ± 0.6 R^2 for visualization purposes. Each separate model was evaluated using 5-fold CV. One marker represents the mean score for one protein using different supervised learning algorithms. The hyper-parameters for each model were tuned on the first example protein in the data-set (A5Z2X5/Min8/YPR010C-A) with cross-validated bayesian optimization, using sci-kit optimize (v0.9.0)

7 Supplementary Notes VII (Representation comparison)

To ensure that the pattern-search did not result in any significant loss of information for a subset of proteins, the relational data present in the Datalog-database was directly instantiated. This resulted in a very high-dimensional data-set (similar to a binary relation matrix, a sparse matrix connecting each observation with all of its relations) representing all of the relations present in the database. Each row in the matrix corresponds to a gene, and each column corresponds to a functional or phenotypical attribute of that gene. An existing relation between an entity (gene) and an attribute is represented by a 1. This matrix was then used as training data for an ElasticNet algorithm, where the protein abundances (for each entity) were used as the predictive target.

This instantiation will contain more information across the whole set of proteins (albeit losing some expressiveness), as it is an explicit representation of the database not bounded by the level-wise search performed by the WARMR-algorithm. Do note however, that this sacrifices a high degree of specific interpretability as it will be very difficult to extract the patterns found in the relational representation.

Note that XGBoost was not selected for comparative purposes due to the significant increase in computational cost (caused by the extremely high-dimensional space provided by the instantiated features). ElasticNet was instead selected due to comparative results in benchmarking (see Supplementary Figures 2 and 3) and its ability to efficiently deal with linear high dimensional learning problems (due to its use of L1 regularization).

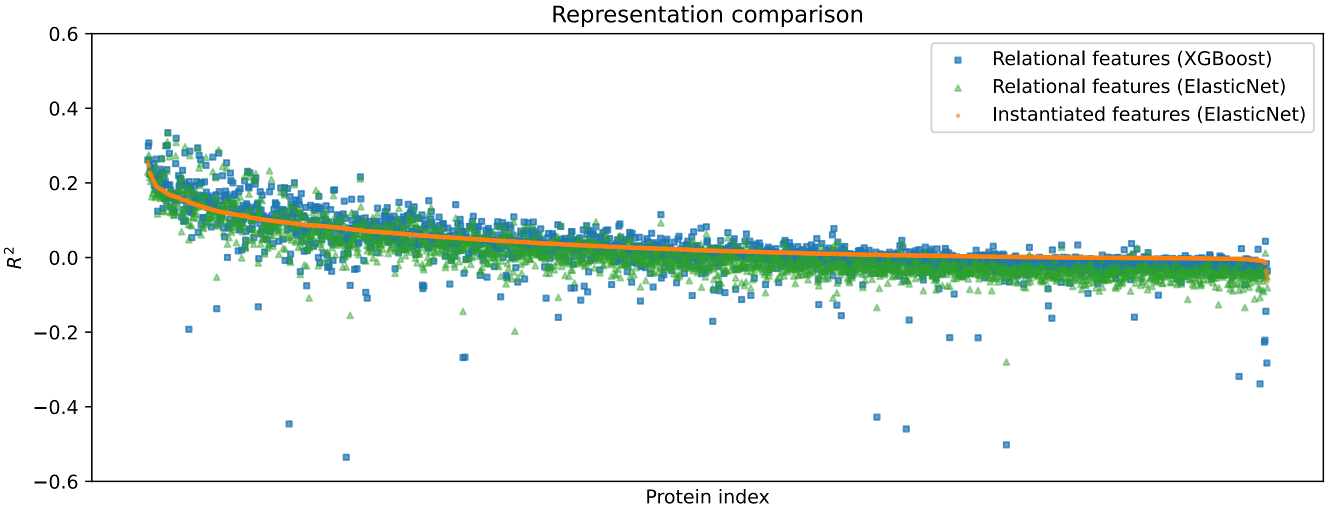

Figure 4: Representation comparison between the generated relational features and an explicit representation of the database. Visualized is protein abundance predictability according to mean R^2 across 2292 proteins in the data-set. The plot was truncated at ± 0.6 R^2 for visualization purposes. Sorted in descending order according to mean R^2 for the instantiated representation. Each separate model was evaluated using 5-fold CV. One marker represents the mean score for one protein using the different representations. The hyper-parameters for each model were tuned on the first example protein in the data-set (A5Z2X5/Min8/YPR010C-A) with cross-validated bayesian optimization, using sci-kit optimize (v0.9.0).

8 Supplementary Notes VIII (Over-representation tests)

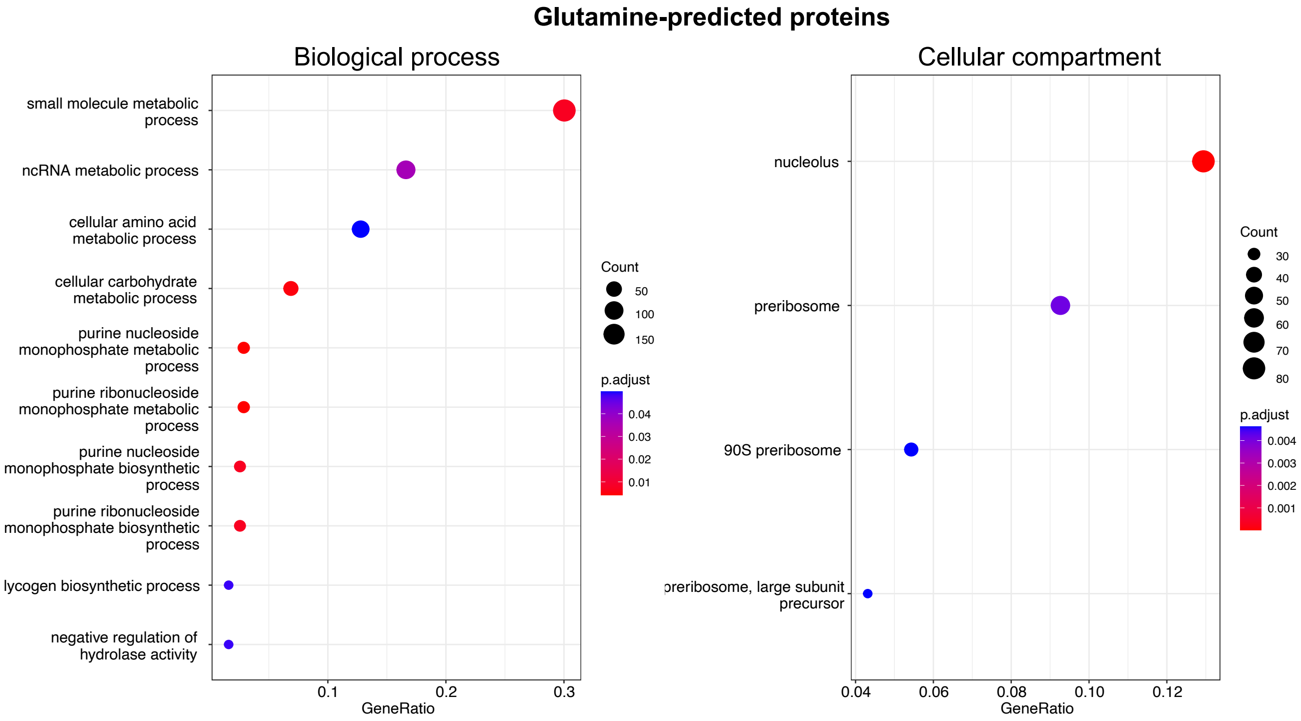

Figure 5: GO over-representation-test (Adj. P-value < 0.05, one-sided Fisher's exact test, adjusted with Benjamini-Hochberg) for the three main GO-categories, using ClusterProfiler. Enrichment is done for proteins primarily predicted by glutamine, using the set of 1640 predictable proteins ($R^2 > 0.0$) as the background. No significant terms found on molecular function.

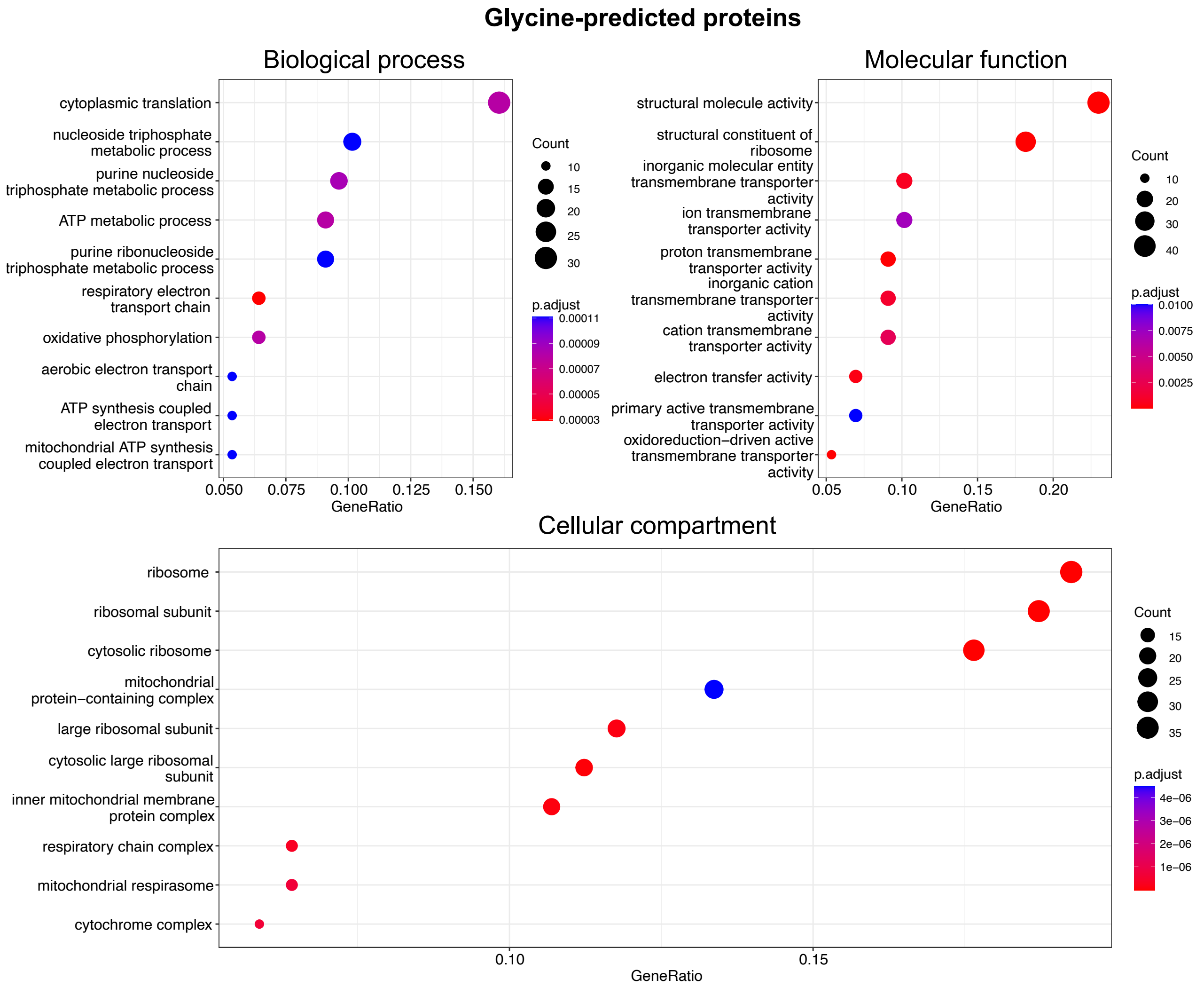

Figure 6: GO over-representation-test (Adj. P-value < 0.05, one-sided Fisher's exact test, adjusted with Benjamini-Hochberg) for the three main GO-categories, using ClusterProfiler. Enrichment is done for proteins primarily predicted by glycine, using the set of 1640 predictable proteins ($R^2 > 0.0$) as the background.

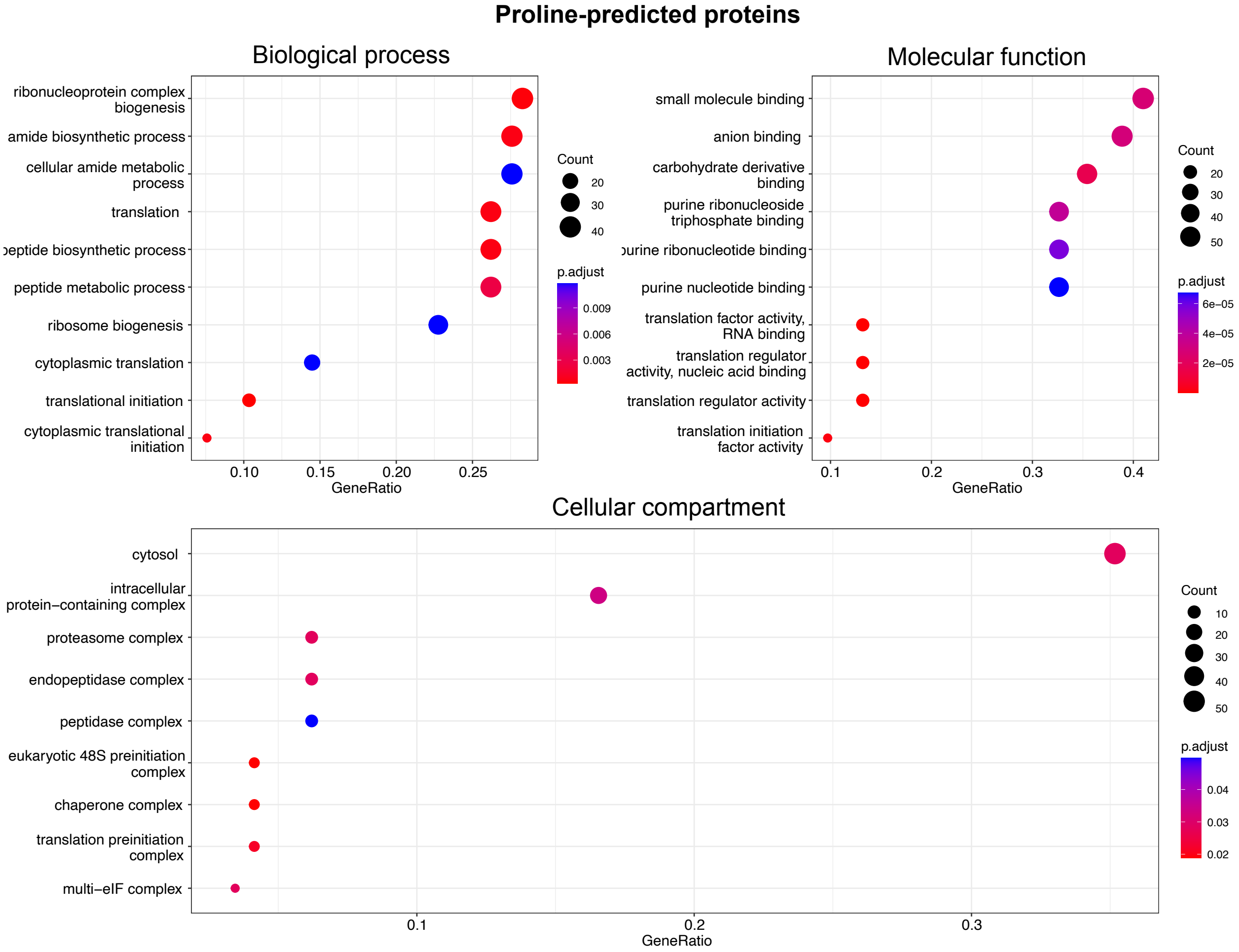

Figure 7: GO over-representation-test (Adj. P-value < 0.05, one-sided Fisher's exact test, adjusted with Benjamini-Hochberg) for the three main GO-categories, using ClusterProfiler. Enrichment is done for proteins primarily predicted by proline, using the set of 1640 predictable proteins ($R^2 > 0.0$) as the background.

References

[1] Hongzhong Lu, Feiran Li, Benjamín J. Sánchez, Zhengming Zhu, Gang Li, Iván Domenzain, Simonas Marcišauskas, Petre Mihail Anton, Dimitra Lappa, Christian Lieven, Moritz Emanuel Beber, Nikolaus Sonnenschein, Eduard J. Kerkhoven, and Jens Nielsen. A consensus S. cerevisiae metabolic model Yeast8 and its ecosystem for comprehensively probing cellular metabolism. *Nature Communications*, 10(1):3586, August 2019.

[2] Rama Balakrishnan, Julie Park, Kalpana Karra, Benjamin C. Hitz, Gail Binkley, Eurie L. Hong, Julie Sullivan, Gos Micklem, and J. Michael Cherry. YeastMine—an integrated data warehouse for Saccharomyces cerevisiae data as a multipurpose tool-kit. *Database: The Journal of Biological Databases and Curation*, 2012:bar062, February 2012.

[3] Michael Ashburner, Catherine A. Ball, Judith A. Blake, David Botstein, Heather Butler, J. Michael Cherry, Allan P. Davis, Kara Dolinski, Selina S. Dwight, Janan T. Eppig, Midori A. Harris, David P. Hill, Laurie Issel-Tarver, Andrew Kasarskis, Suzanna Lewis, John C. Matese, Joel E. Richardson, Martin Ringwald, Gerald M. Rubin, and Gavin Sherlock. Gene Ontology: tool for the unification of biology. *Nature genetics*, 25(1):25–29, May 2000.
